# Vasorin deficiency leads to cardiac hypertrophy by targeting MYL7 in young mice

**DOI:** 10.1111/jcmm.17034

**Published:** 2021-12-02

**Authors:** Junming Sun, Xiaoping Guo, Ping Yu, Jinning Liang, Zhongxiang Mo, Mingyuan Zhang, Lichao Yang, Xuejing Huang, Bing Hu, Jiajuan Liu, Yiqiang Ouyang, Min He

**Affiliations:** ^1^ Laboratory Animal Center Guangxi Medical University Nanning Guangxi China; ^2^ Department of Cardiology The Second Affiliated Hospital Guangxi Medical University Nanning Guangxi China; ^3^ School of Public Health Guangxi Medical University Nanning China; ^4^ Ministry of Education Key Laboratory of High‐Incidence‐Tumor Prevention & Treatment, (Guangxi Medical University) Nanning China

**Keywords:** cardiac hypertrophy, deficiency, MYL7, Vasorin

## Abstract

Vasorin (VASN) is an important transmembrane protein associated with development and disease. However, it is not clear whether the death of mice with VASN deficiency (*VASN* ^−/−^) is related to cardiac dysfunction. The aim of this research was to ascertain whether VASN induces pathological cardiac hypertrophy by targeting myosin light chain 7 (MYL7). *VASN* ^−/−^ mice were produced by CRISPR/Cas9 technology and inbreeding. PCR amplification, electrophoresis, real‐time PCR and Western blotting were used to confirm VASN deficiency. Cardiac hypertrophy was examined by blood tests, histological analysis and real‐time PCR, and key downstream factors were identified by RNA sequencing and real‐time PCR. Western blotting, immunohistochemistry and electron microscopy analysis were used to confirm the downregulation of MYL7 production and cardiac structural changes. Our results showed that sudden death of *VASN* ^−/−^ mice occurred 21–28 days after birth. The obvious increases in cardiovascular risk, heart weight and myocardial volume and the upregulation of hypertrophy marker gene expression indicated that cardiac hypertrophy may be the cause of death in young *VASN* ^−/−^ mice. Transcriptome analysis revealed that VASN deficiency led to MYL7 downregulation, which induced myocardial structure abnormalities and disorders. Our results revealed a pathological phenomenon in which VASN deficiency may lead to cardiac hypertrophy by downregulating MYL7 production. However, more research is necessary to elucidate the underlying mechanism.

## 1 INTRODUCTION

Vasorin (VASN), also known as SLIT‐like 2 (slitl2), is a 673‐amino acid transmembrane glycoprotein localized on the cell surface^1^ that is cleaved by a disintegrin and metalloproteinase. VASN is considered a potential biomarker and therapeutic target for cancer.^2^, ^3^ Notably, VASN is highly conserved in many organs from embryonic development to adulthood.^4^, ^5^ There is limited literature on VASN function. In embryonic development, *VASN* is primarily expressed in the heart and lungs.^1^ *VASN* is expressed in vascular smooth muscle cells^1^ and umbilical vein endothelial cells.^6^ Soluble VASN protein can bind to TGF‐β, which can inhibit epithelial‐to‐mesenchymal transition.^7^ A previous study showed high VASN expression in human glioblastoma in the context of TNF α‐induced apoptosis.^7^ CRISPR/Cas9 technology could be used to generate a knockout mouse model to further study the function of VASN and its associated signalling pathway. However, unexplained death occurs in *VASN* ^−/−^ mice shortly after birth,^7^ and it was unclear whether this phenomenon was related to heart damage.

Cardiac hypertrophy may lead to heart damage and sudden death^8^, ^9^, ^10^ but is not always pathological (ie associated with cardiac dysfunction). Cardiac hypertrophy is not present under conditions of increased chamber volume but normal cardiac mass. Pathological cardiac hypertrophy is a compensatory change in cardiac overload induced by pathological stimulation^11^, ^12^ that is characterized by increased heart weight or volume, myocardial cell volume and extracellular matrix,^13^, ^14^properties of the inevitable process by which heart disease progresses to heart failure.^15^, ^16^ The pathogenesis of cardiac hypertrophy is not clear. In a recent study, monogenic mutation of cardiac sarcomeres was shown to cause pathological cardiac hypertrophy.^17^, ^18^ Cardiac myosin is an important structure in cardiac sarcomeres and includes two myosin regulatory light chains (MLC2v and MLC2a).^19^ Myosin light chain 7 (*MYL7*), also known as MLC2a, is expressed in heart ventricles and atria.^20^, ^21^ *MYL7* inactivation leads to embryonic lethality and abnormal cardiac morphogenesis.^22^, ^23^ Based on these studies, a novel pathological phenomenon was hypothesized in which *VASN* deletion leads to cardiac hypertrophy by affecting *MYL7* expression.

In this study, we used CRISPR/Cas9 technology to produce *VASN*‐knockout mice and investigated the changes in cardiac function and cardiac hypertrophy index values. Our results indicated that cardiac hypertrophy may be the cause of death in young *VASN*‐knockout mice. We found that VASN deficiency downregulated *MYL7* expression, resulting in myocardial structure abnormalities and disorders. Our results reveal a pathological phenomenon in which VASN deficiency may lead to cardiac hypertrophy by downregulating MYL7 production.

## 2 MATERIALS AND METHODS

### 2.1 Mouse lines and embryo manipulation

All mouse experiments were approved by the ethics committee of Guangxi Medical University. C57BL/6J mice were obtained from the Laboratory Animal Center of Guangxi Medical University [syxk GUI 2020–0004]. Embryonic manipulation was carried out on our custom embryo platform.^24^ C57BL/6J females were subjected to oestrus synchronization and superovulation.^25^ Eight females were mated with eight fertile males each time, and the pronucleus was injected with approximately 10 pL mRNA. Approximately forty injected embryos were transferred into the fallopian tube of each pseudopregnant mouse.

### 2.2 Production of *VASN*‐knockout mouse

*VASN*‐knockout mice were created by CRISPR/Cas9 technology.^26^ The *VASN* gene (GenBank accession number: NM_139307.3; Ensembl: ENSMUSG00000039646) is located on mouse chromosome 16. Two pairs of guide RNA (gRNA) were constructed and confirmed by sequencing (Table 1). Cas9 mRNA and gRNA were coinjected into the pronucleus to produce *VASN* ^−/+^ mice. *VASN* ^−/−^ mice were produced by inbreeding eight pairs of 8 ‐ to 9 ‐week‐old *VASN* ^−/+^ females and males. *VASN* ^+/+^, *VASN* ^−/+^ and *VASN* ^+/+^ mice were divided into three groups.

**TABLE 1:** List of primer sequences

Genes	Forward/ Reverse	Sequences
gRNA1	‐	TCCCCAGACGGGGACCCGACAGG
gRNA2	‐	GTACTGGATGCACTGGCGGCTGG
gRNA3	‐	ATGTGATCATACCACGACGGTGG
gRNA4	‐	TTCCCTTCAGGGGCGAGACCTGG
P1	‐	ACTTCTGGGGGACAATGCTG
P2	‐	ACTGGAGTTTCTTG CCTTGGT
P3	‐	GCCTCAACAGTCAGCTTCTC
P4	‐	CTGATGGGCACCCTGTACT
VASN	Forward Reverse	CCGGGACCCCGGACCTCTCA TCTTCTGTCCCAGGAGACGACTGCG
BNP	Forward Reverse	TTCTGCTCCTGCTTTTCCTT GCCATTTCCTCTGACTTTTC
MYH7	Forward Reverse	GATGGTGACACGCATCAACG CCATGCCGAAGTCAATAAACG
MYL7	Forward Reverse	CTCATGACCCAGGCAGACAAG CCGTGGGTGATGATGTAGCAG
UCP1	Forward Reverse	CTCAGCCGGAGTTTCAGCTTG GAAGCCTGGCCTTCACCTTG
A2 M	Forward Reverse	CATTTGCTTTGGTGGTGCAGA TCAGCAATTGCCATGTTGGAG
MYL4	Forward Reverse	CCAATGGCTGCATCAACTATGAA CCATGTGAGTCCAATACTCCGTAA
CACNA1D	Forward Reverse	TGCAAGATGACGAGCCAGAAG TGATTGACATGGTTTCCAAGCAG

### 2.3 PCR amplification and electrophoresis

Each mouse genotype was identified by PCR amplification and electrophoresis.^27^ DNA was extracted according to the manufacturer's instructions (TransGen, China). DNA polymerase (Vazyme, China) and two primer pairs (Table 1) were used for PCR amplification under the following amplification conditions: 95 °C for 3 min; 35 cycles of 95 °C for 15 s, 60 °C for 15 s and 72 °C for 2.5 min; and 72 °C for 5 min. PCR products were visualized by 1.5% agarose gel electrophoresis (Invitrogen, USA).

### 2.4 Cardiovascular index analysis

The mice in each group (3 mice, 2 biological replicates per group) were anaesthetized with 4% chloral hydrate (0.02 mL g^−1^). Approximately 400 μL of blood was collected from each mouse and placed at room temperature for 30 min. The resulting supernatant was stored at −20°C. Serum homocysteine (HCY) levels were measured by an AEROSET‐2 000 automatic analyser (Abbott, USA). The levels of serum cardiac enzymes (lactate dehydrogenase (LDH) and creatine kinase (CK)) and myocardial enzymes (creatine kinase isoenzyme (CK‐MB)) were measured by an AU5800 automatic analyser (Beckman Coulter, USA).

### 2.5 Pathological analysis

Haematoxylin and eosin (HE) staining was performed as previously described.^28^ Pathological changes in the heart were detected in mice from each group (3 mice, 5 biological replicates per group). The percentage of mice with cardiac hypertrophy was calculated as the number of mice with cardiac hypertrophy divided by the total number of mice in each group.

### 2.6 Transcriptome sequencing

Transcriptome sequencing of heart tissue from the three groups was completed at the Wuhan Genome Institute (BGI‐Shenzhen). Twelve total RNA samples (4 mice per group) were sequenced. Whole transcriptome data were collected and compared with the ribosome database to identify known transcripts (mRNA), perform quantitative analysis of known and new mRNAs, analyse differences between samples (at least 2 samples) and analyse differences between groups (at least 2 samples and at least 3 biological replicates per group). Differentially expressed genes were analysed using the DAVID database by identifying Gene Ontology (GO) terms and Kyoto Encyclopedia of Genes and Genomes (KEGG) pathway maps.

### 2.7 Real‐time quantitative PCR analysis

RNA was extracted from heart tissue from each group (3 samples, 3 biological replicates per group) as previously described.^29^ Reverse transcription and quantitative PCR were carried out in accordance with a previous article.^30^ Primers (Table 1) were produced by Sangon Biotech (Shanghai). Each gene was subjected to 40 cycles of PCR, and this process was repeated three times. Expression of the endogenous gene 18S was used for comparison. The relative expression levels of target genes were calculated by using the 2^–△△CT^ method.

### 2.8 Western blotting analysis

Hearts from mice in each group (2 samples, 4 biological replicates per group) were prepared as previously described.^31^ Primary antibodies against MYL7 (17283–1‐AP, Proteintech, 1:2000), VASN (A16215, ABclonal, 1:1000) and GAPDH (AC001, ABclonal, 1:1000) were used, and the secondary antibody was horseradish peroxidase (HRP)‐conjugated goat anti‐rabbit antibody (AS014, ABclonal, 1:1000). Protein expression was calculated by using an automatic analysis system (Image Lab 6.0). The target protein data were normalized to GAPDH data.

### 2.9 Immunohistochemistry analysis

Hearts from mice in each group (4 samples, 4 biological replicates per group) were prepared as previously described.^32^ The MYL7 primary antibody (17283–1‐AP, Proteintech, 1:1000) and HRP secondary antibody (AS014, ABclonal, 1:1000) were used. Relative MYL7 expression was calculated as integrated optical density (IOD)/area by using a quantitative digital image analysis system (Image‐Pro Plus 6.0).

#### 2.9.1 Electron microscopy analysis

The isolated hearts from mice in each group (2 samples, 2 biological replicates per group) were prepared as previously described.^33^ Heart tissues were fixed in 5% glutaraldehyde for 2 h and 2% osmium tetroxide for 3 h. Next, the tissue samples were immersed in an alcohol gradient (30, 50, 70, 90, 95 and 100%) for 10 min at each percentage, 100% alcohol for another 30 min and anhydrous propylene oxide for 10 min; then, the tissues were immersed in epoxy propane (acetone) and epoxy resin at a ratio of 3:1, 1:1 and 1:3 for 3 h each and, finally, in epoxy resin for 12 h. A catalyst (2%) was added to the epoxy resin, and the samples were baked at 35°C for 12 h, 45°C for 12 h and 60°C for 24 h. The samples were observed under an electron microscope (Thermo). Three random fields of each sample were photographed to count the number of damaged mitochondria and assess myocardial injury.

#### 2.9.2 Statistical analysis

All results are shown as the mean and standard deviation. Data were analysed by Duncan's multiple comparison. *p* < 0.05 was considered indicative of a significant difference. *p* < 0.01 was considered highly significant.

## 3 RESULTS

### 3.1 Generation of *VASN* ^−/−^ mice using CRISPR/Cas9 technology

**FIGURE 1:**
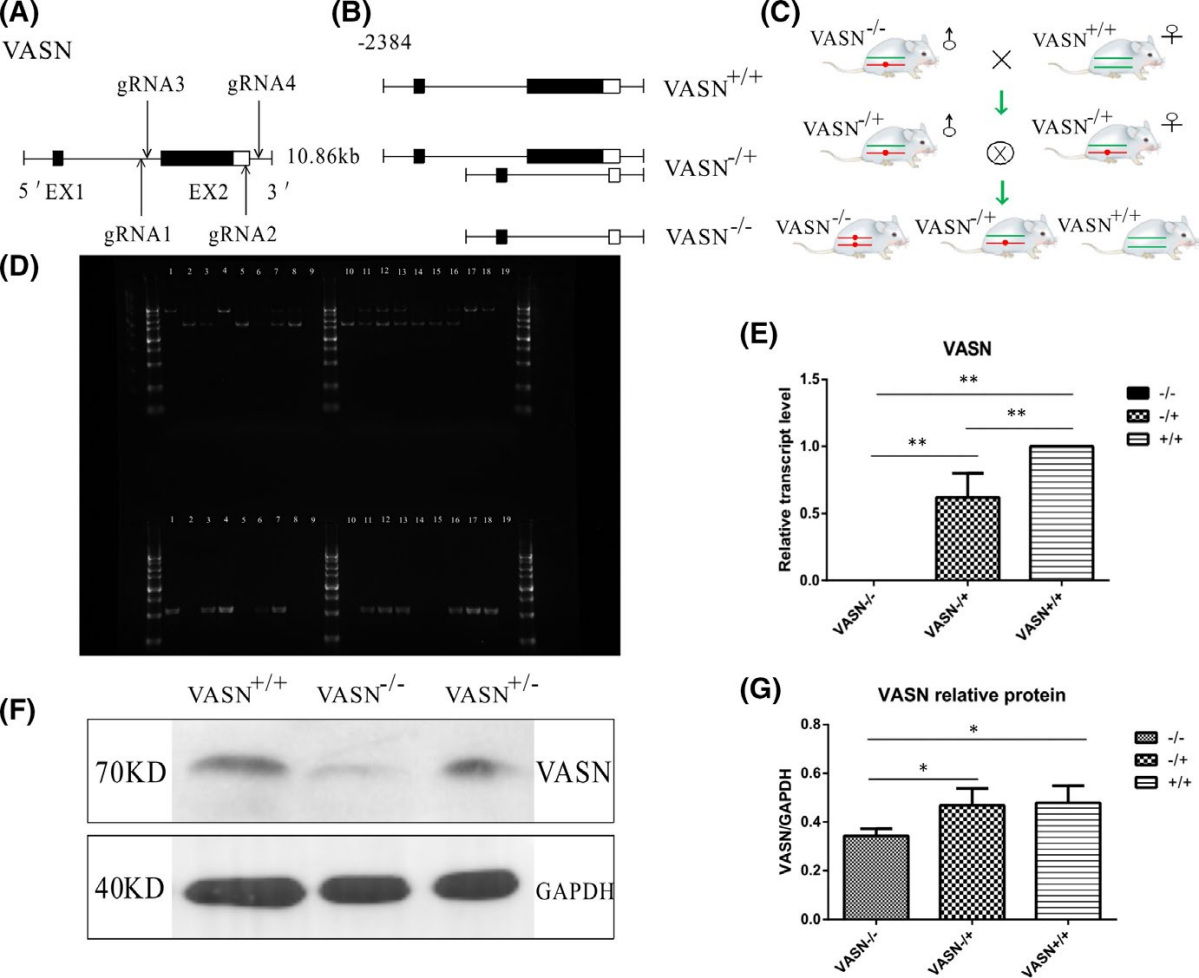
Characterization of VASN knockout in C57BL/6J mice. (A) *VASN*‐knockout rule. (B) Criteria for *VASN* knockout: *VASN* ^−/−^ gene, 1600 bp; *VASN* ^−/+^ gene, 4 000 bp, 1600 bp and 800 bp; *VASN* ^+/+^ gene, 4 000 bp and 800 bp. (C) *VASN* ^−/−^ mice production process. (D) Electrophoretogram of *VASN* DNA analysis: lanes 2, 5, 8, 10, 14 and 15, *VASN* ^−/−^ DNA; 3, 7, 11, 12, 13 and 16, *VASN* ^−/+^ DNA; 1, 4, 17 and 18, *VASN* ^+/+^ DNA; 9 and 19, negative control. (E) Quantitation of the relative expression of *VASN*. (F) Changes in VASN protein expression. (G) Quantitation of the relative expression of VASN protein. ^*^ *p* < 0.05 and ^**^ *p* < 0.01 by one‐way ANOVA

The coding region of mouse *VASN* has two exons, 1 and 2, of which exon 2 is the main transcription region. We generated *VASN* heterozygous mice by knockout of exon 2 using CRISPR/Cas9 technology (Figure 1A). We established a heterozygous mouse line with a stable deletion of 2384bp in the targeted *VASN* site (Figure 1B). We produced homozygous (*VASN* ^−/−^) and heterozygous *VASN*‐knockout (*VASN* ^−/+^) and wild‐type (*VASN* ^+/+^) mice by inbreeding of *VASN* ^−/+^mice (Figure 1C). The mouse genotypes were identified by PCR amplification and electrophoresis (Figure 1D). VASN mRNA and protein levels were dramatically reduced in *VASN* ^−/−^ mice compared with *VASN* ^−/+^ and *VASN* ^+/+^ mice (Figure 1E, 1F‐1G).

### 3.2 *VASN*‐knockout results in premature death and cardiovascular risk in young mice

**FIGURE 2:**
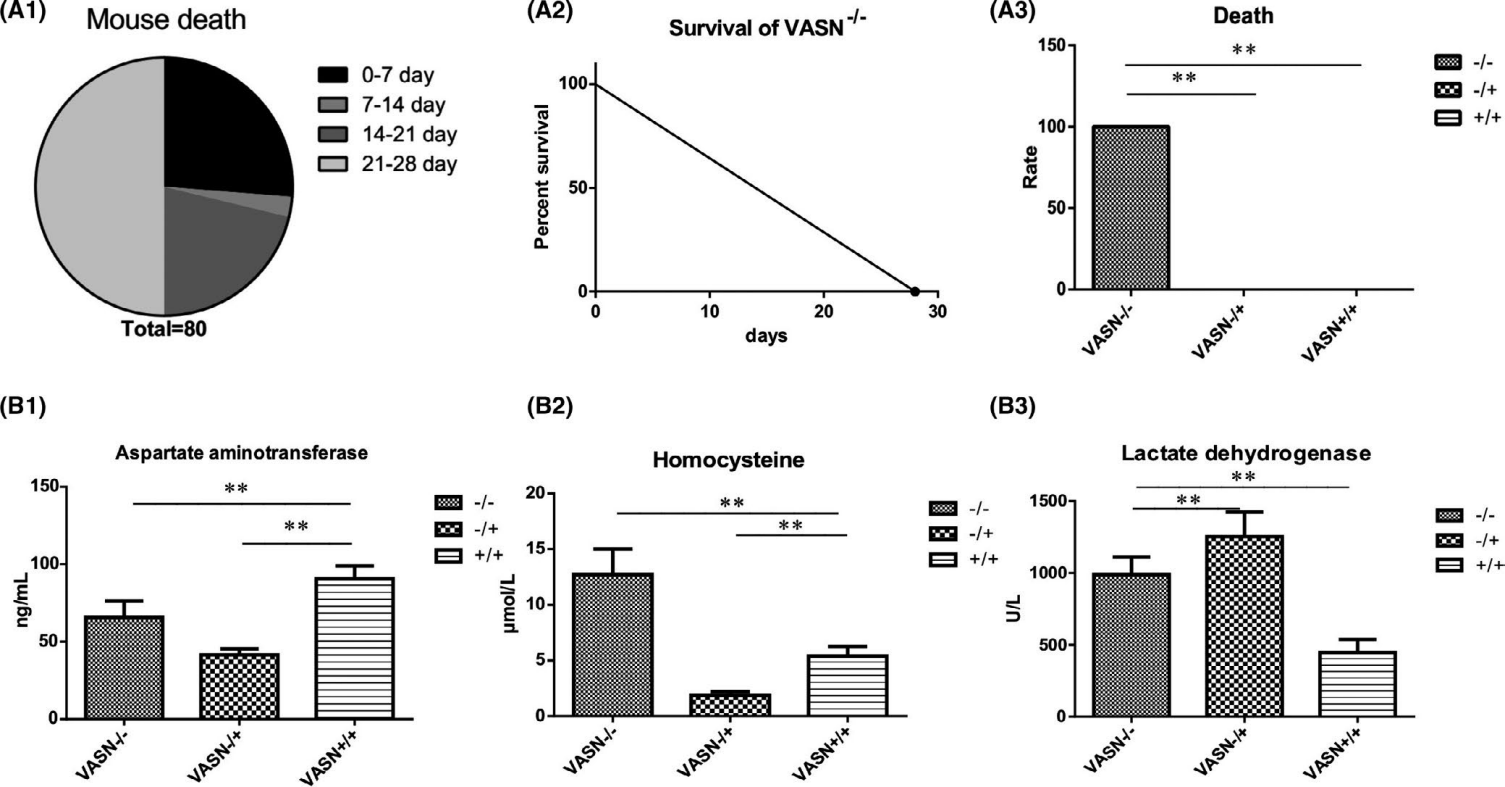
Survival and cardiovascular risk in young mice. (A1) Distribution of time of death among *VASN* ^−/−^mice. (A2) Changes in the relationship between death and time in *VASN* ^−/−^ mice. (A3) Death rate of different *VASN*‐knockout mice over 28 days. (B1‐B3) Changes in cardiovascular risk factors (aspartate aminotransferase, homocysteine and lactate dehydrogenase levels) in different *VASN*‐knockout mice. ^**^ *p* < 0.01 by one‐way ANOVA

To observe the effect of *VASN* knockout on mice, we analysed the survival time of mice with different genotypes. *VASN* ^−/−^ mice died by 28 days, and half of them died 21–28 days after birth (Figure 2A1). The survival rate of *VASN* ^−/−^ mice sharply decreased over time since birth (Figure 2A2). Premature death was not observed in *VASN* ^−/+^ and *VASN* ^+/+^ mice (Figure 2A3). Aspartate aminotransferase (AST) levels were significantly decreased in *VASN* ^−/−^ mice compared with *VASN* ^+/+^ mice (Figure 2B1), whereas HCY and LDH levels were significantly increased in *VASN* ^−/−^ mice compared with *VASN* ^+/+^ mice Figure 2B1‐B2) In addition, *VASN* ^−/−^ mice were observed to be listless and wobbly. These results suggest that heart pathology may be the cause of sudden death in young *VASN* ^−/−^ mice.

### 3.3 *VASN* deficiency leads to cardiac hypertrophy in young mice

**FIGURE 3:**
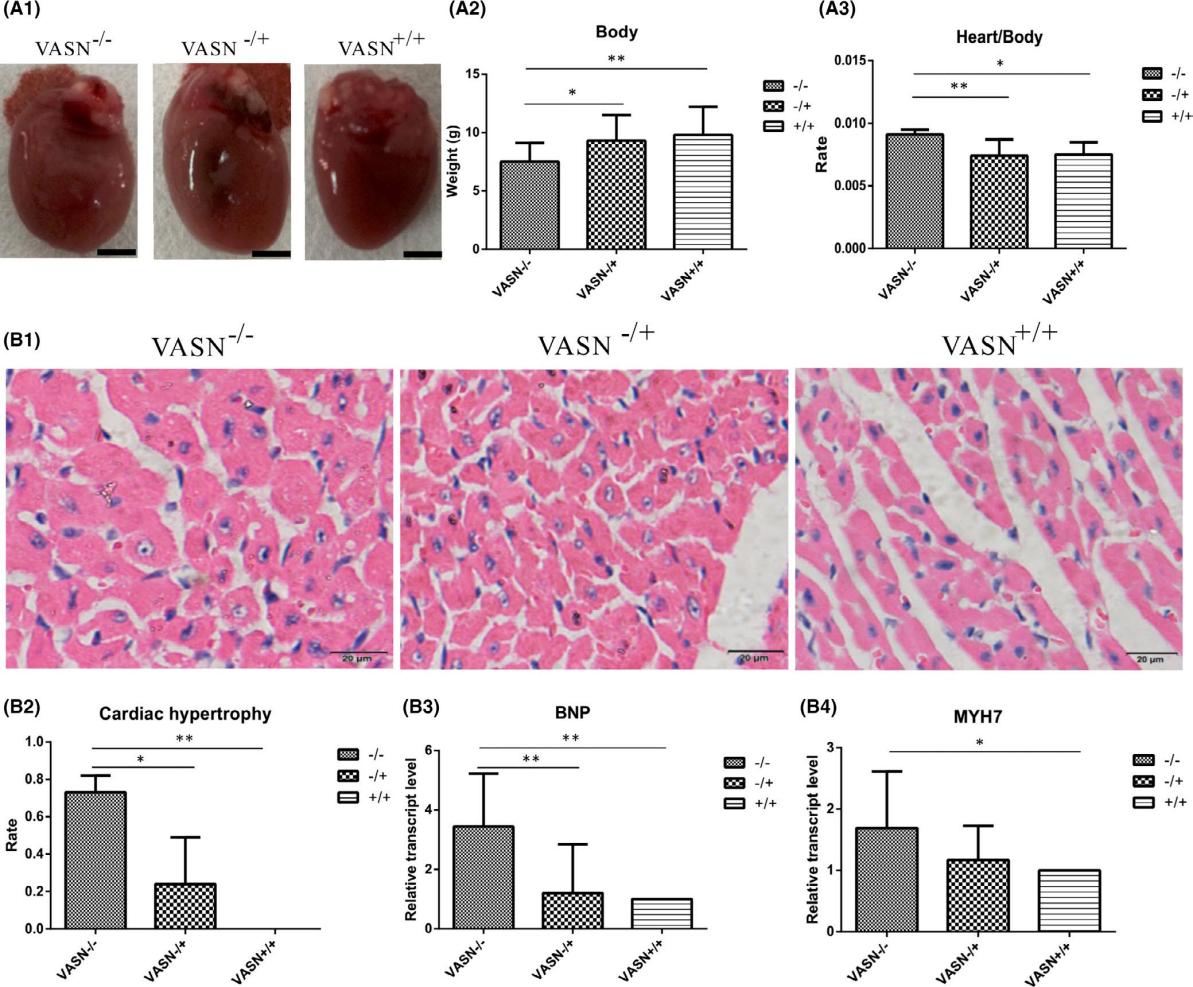
Typical pathological indexes of cardiac hypertrophy in young mice. (A1) Hearts from different *VASN*‐knockout mice; Scale bar, 5 mm. (A2) Changes in body weight of different *VASN*‐knockout mice. (A3) Changes in heart weight/body weight ratio of different *VASN*‐knockout mice. (B1) HE staining showing pathological changes in cardiac hypertrophy in different *VASN*‐knockout mice. (B2) The rate of cardiac hypertrophy in different *VASN*‐knockout mice. (B3‐B4) Quantitation of the relative expression of cardiac hypertrophy marker genes (*BNP* and *MYH7*) in different VASN‐knockout mice. ^*^ *p* < 0.05 and ^**^ *p* < 0.01 by one‐way ANOVA

To explore whether the cause of death of *VASN* ^−/−^ mice is related to the heart, we detected pathological changes in this organ. *VASN* ^−/−^mice exhibited increased heart volume (Figure 3A1), reduced body weight (Figure 3A2)) and an increased heart weight/body weight ratio compared with *VASN* ^+/+^ mice (Figure 3A3)). HE staining showed that cardiomyocyte volume was significantly larger in *VASN* ^−/−^ mice than in *VASN* ^−/+^ and *VASN* ^+/+^ mice (Figure 3B1). Approximately 70% of *VASN* ^−/−^ mice had cardiac hypertrophy; this percentage was higher than that in *VASN* ^−/+^ and *VASN* ^+/+^ mice (Figure 3B2). The expression of cardiac hypertrophy marker genes (*BNP* and *MYH7)* was significantly upregulated in *VASN* ^−/−^ mice compared with *VASN* ^+/+^ mice (Figure 3B3‐B4). These results show that *VASN* deficiency induces cardiac hypertrophy in young mice.

### 3.4 *VASN* deficiency reduces *MYL7* expression in young mice

**FIGURE 4:**
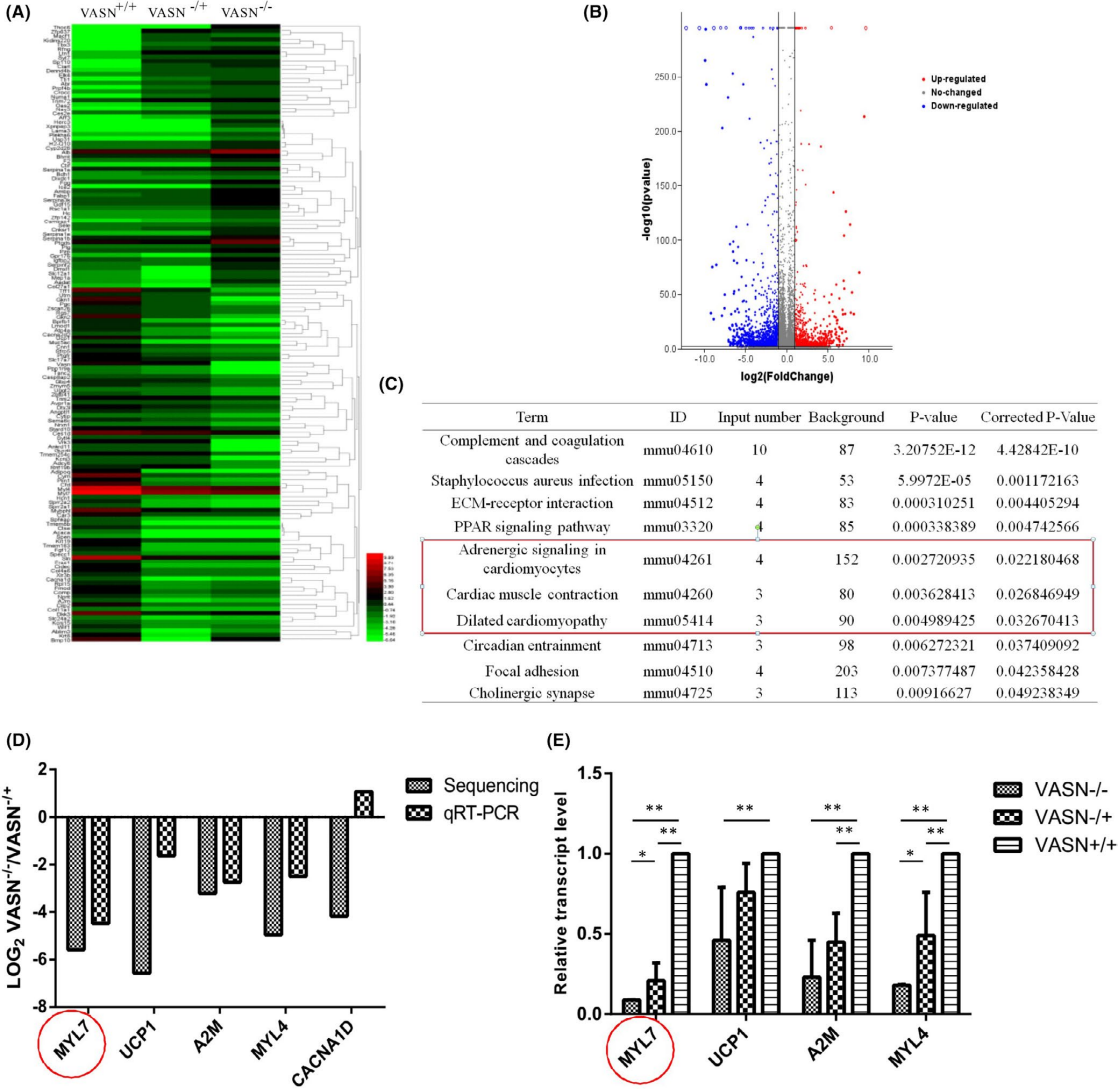
Sequencing and quantitation of the relative expression of related genes in young *VASN*‐knockout mice. (A) Hotspot map. (B) Volcano map. (C) Signalling pathways identified by KEGG and GO analyses. The box indicates that the signalling pathway contains a common *MYL7* gene. (D) Quantitative verification of the relative expression of enriched genes. (E) Quantitative verification of the relative expression of *UCP1*, *MYL7*, *A2 M* and *MYL4*. ^*^ *p* < 0.05 and ^**^ *p* < 0.01 by one‐way ANOVA

To investigate the changes in potential downstream genes caused by *VASN* deficiency, we performed transcriptome analysis of hearts from *VASN* ^−/−^, *VASN* ^−/+^ and *VASN* ^+/+^ mice using RNA sequencing. According to standard expression value ≥100 and log absolute value ≥2, we identified 61 upregulated genes and 82 downregulated genes (Figure 4A‐4B). *VASN* was among the most drastically downregulated genes. KEGG analysis revealed that *VASN* deficiency affected signalling pathways (Figure 4C). We selected genes for qRT‐PCR verification and validated *MYL7* among the genes following the selected trend with a high correlation (Figure 4D). Real‐time PCR analysis further confirmed that *MYL7* expression in the heart was significantly downregulated in *VASN* ^−/−^ mice compared to *VASN* ^−/+^ and *VASN* ^+/+^ mice (Figure 4E). These data suggest that *VASN* deficiency regulates *MYL7* expression.

### 3.5 *VASN* deficiency induces cardiac hypertrophy by downregulating *MYL7* expression

**FIGURE 5:**
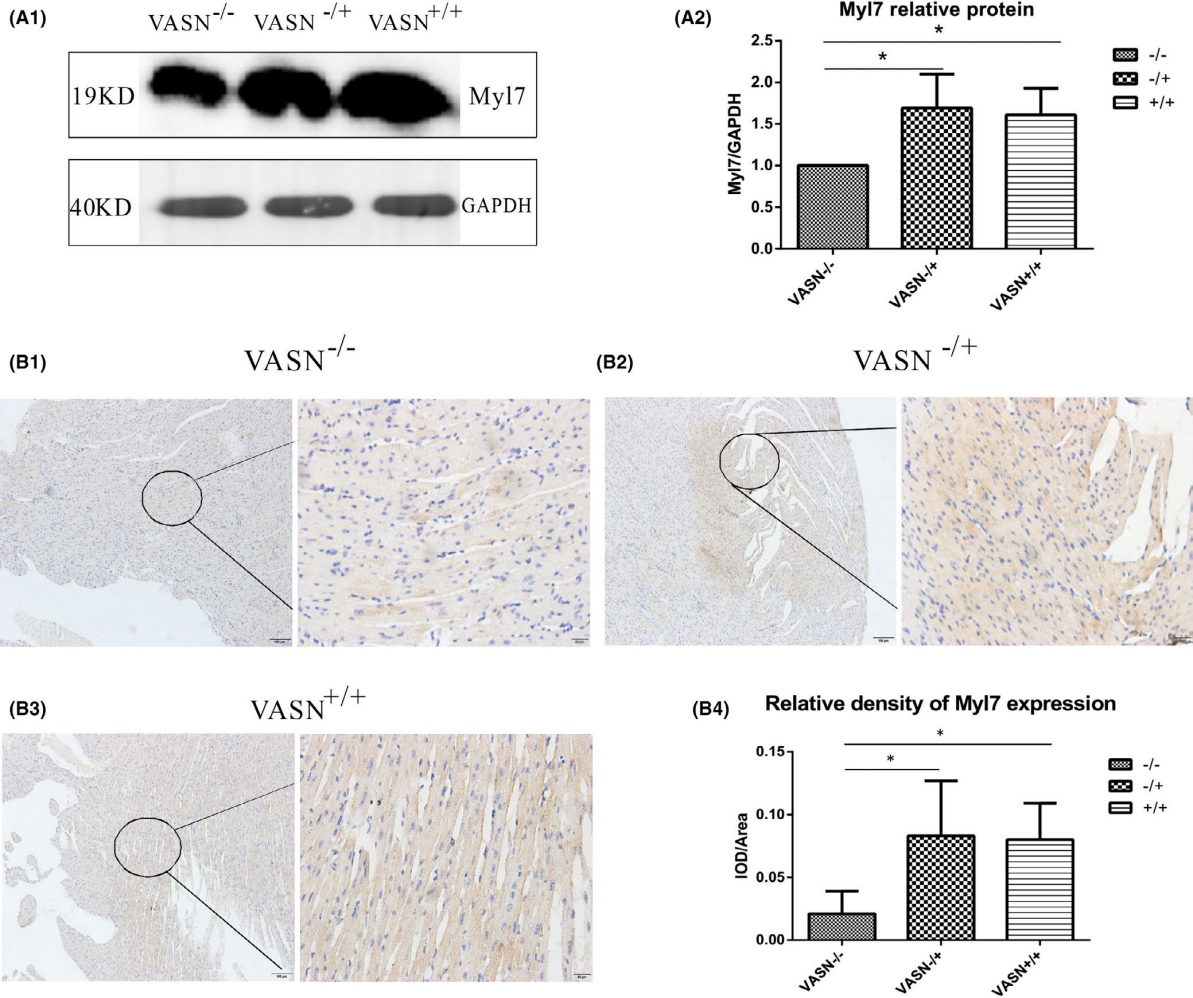
Changes in MYL7 protein in young *VASN*‐knockout mice. (A1) Changes in MYL7 protein expression. (A2) Quantitation of changes in the relative expression of MYL7 protein. (B1‐B3) Immunohistochemical analysis of MYL7. (B4) Changes in the relative density of MYL7. ^*^ *p* < 0.05 and ^**^ *p* < 0.01 by one‐way ANOVA

To further study the potential molecular mechanism by which *VASN* deficiency leads to cardiac hypertrophy, we studied whether changes in *MYL7* expression affect myocardial fibre structure. Western blotting analysis showed that MYL7 protein expression was significantly lower in *VASN* ^−/−^ mice hearts than in *VASN* ^−/+^ and *VASN* ^+/+^ mice hearts (Figure 5A1‐A2). The relative density of MYL7 in *VASN* ^−/−^ mice hearts was significantly lower than that in *VASN* ^−/+^ and *VASN* ^+/+^ mice hearts (Figure 5B1‐B4).

**FIGURE 6:**
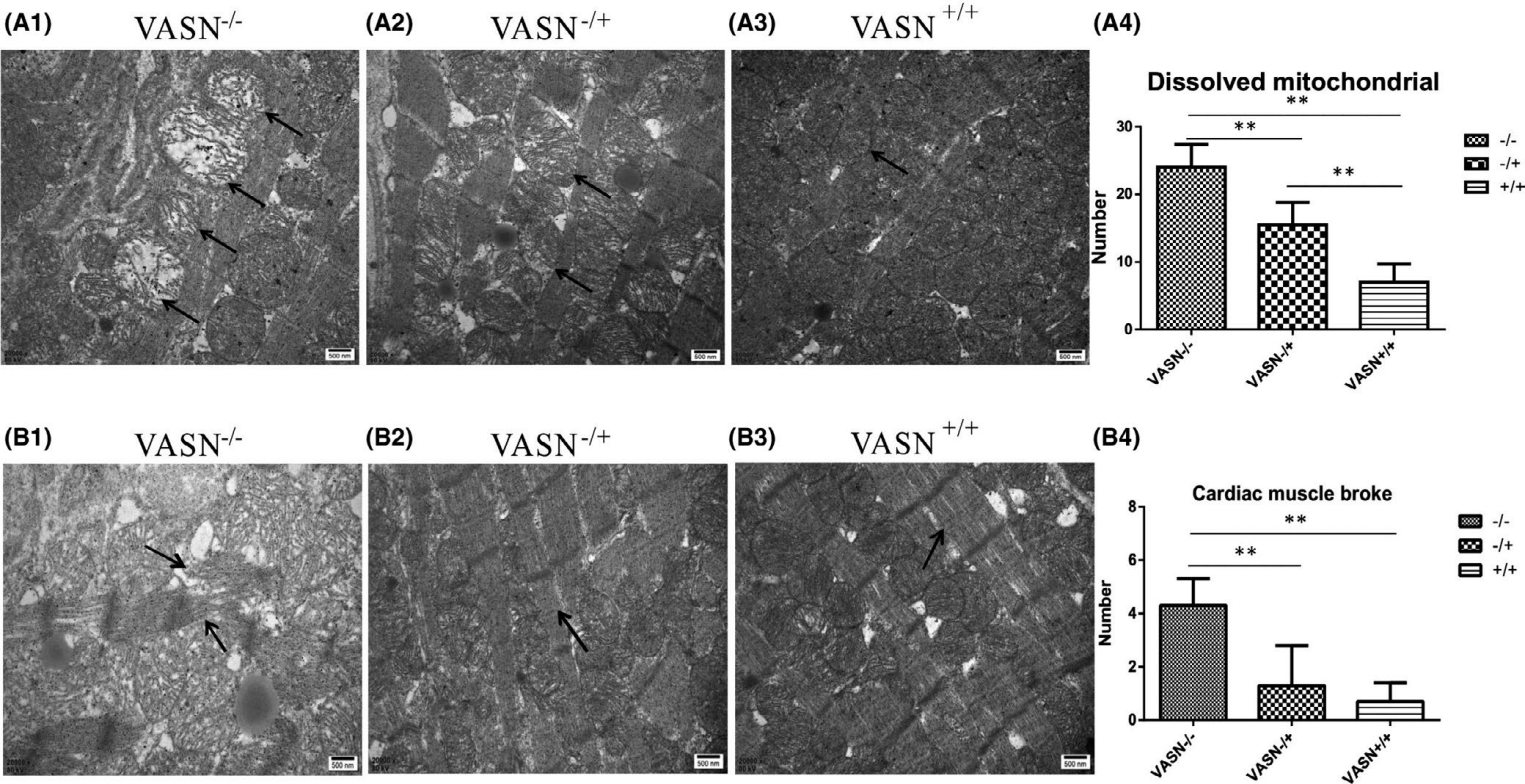
Effect of downregulated *MYL7* expression on myocardial injury in young VASN‐knockout mice. (A1‐A3) Changes in mitochondrial structure were detected with an electron microscope; the arrows indicate mitochondrial structure. The arrows indicate severely damaged mitochondria in the *VASN* ^−/−^ figure, slightly damaged mitochondria in the *VASN* ^−/+^ figure, and normal mitochondria in the *VASN* ^+/+^ Figure 1(A4). Changes in the number of dissolved mitochondria. (B1‐B3) Changes in cardiac muscle breakage induced by *MYL7* downregulation as observed by electron microscopy. The arrow indicates changes in myocardial fibres. The arrow indicates myocardial fibre rupture in the *VASN* ^−/−^figure and normal and continuous myocardial fibres in the *VASN* ^−/+^ and *VASN* ^+/+^ (B4) Changes in the number of broken myocardial fibres. Scale bar, 500 nm. ^*^ *p* < 0.05 and ^**^ *p* < 0.01 by one‐way ANOVA

**FIGURE 7:**
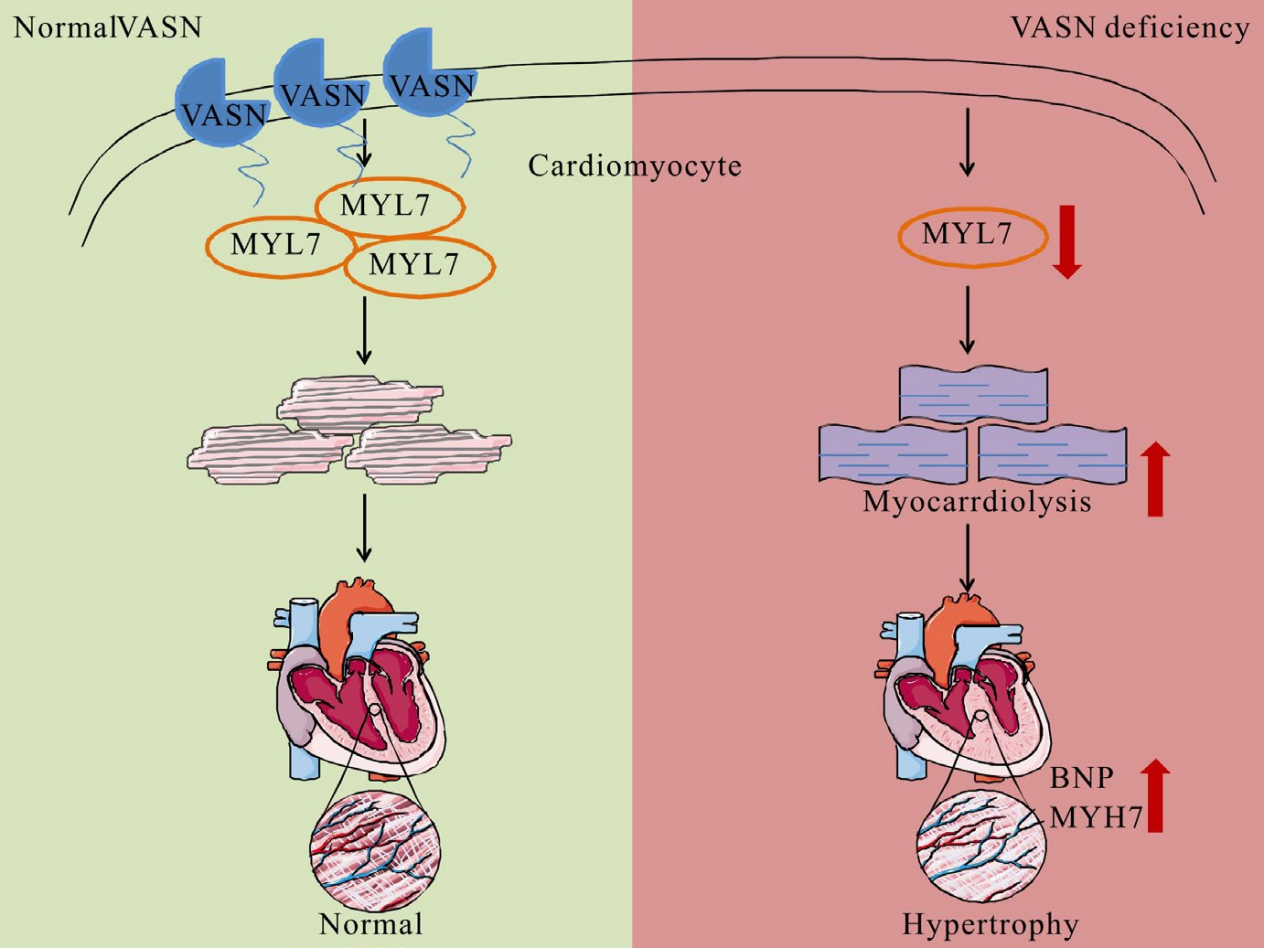
Diagram of the mechanism by which *VASN* deficiency induces cardiac hypertrophy by downregulating *MYL7* expression

In addition, electron microscopy showed that more damage and vacuoles in mitochondria of *VASN* ^−/−^ mice than in those of *VASN* ^−/+^ and *VASN* ^+/+^ mice (Figure 6A1‐A4). Few mitochondrial cristae in *VASN* ^−/+^ mice appeared sparse or destroyed. Mitochondrial crista density, cell membrane integrity, a lack of swelling and intact structures were observed in *VASN* ^+/+^ mice. Myocardial fibres in *VASN* ^−/−^ mice were disordered and irregular, while those in *VASN* ^−/+^ and *VASN* ^+/+^ mice were orderly and regular with clear Z lines (Figure 6B1‐B4). This result implies that the downregulation of MYL7 production causes abnormal myocardial structure. Taken together, these findings show that *VASN* deficiency reduces MYL7 expression, which leads to cardiac hypertrophy (Figure 7, mechanism diagram).

## 4 DISCUSSION

In zebrafish gastrula, *VASN* is diffusely expressed in the brain and central nervous system.^34^ In the early stage of mouse embryonic development, *VASN* is strongly expressed in the hindbrain and neural tube midline.^4^ In adult mice, *VASN* is expressed in the heart, liver, kidney and inner follicle,^1^, ^5^ and in humans, *VASN* is expressed in umbilical vein endothelial cells,^6^ periodontal ligament cells^35^ and follicular fluid.^36^ *VASN* deficiency causes unknown death within 3 weeks.^7^ Our data corresponded with those in previous studies showing sudden death in young *VASN* ^−/−^ mice (Figure 2). Our unpublished studies also showed that *VASN* deficiency induces inflammation and pathology in the lung, kidney and liver. Thus, *VASN* is closely related to the development of various organs and diseases.

The third generation of artificial endonucleases for use in the CRISP/Cas9 system has been successfully developed. Due to its high mutation efficiency, simple production and low cost, this system is considered a molecular tool for genome site‐specific modification with broad application prospects. At present, this technology has been successfully applied to the precise genome modification of human cells,^26^ zebrafish^37^ and mice.^38^ In this study, we used CRISP/Cas9 technology to study VASN function. We generated a 2384‐nucleobase deletion affecting the transcription region that could be inherited stably (Figure 1). Our CRISPR/Cas9 system achieved rapid, efficient and accurate gene editing, as shown in previous studies.

Systemic *VASN* deficiency may lead to pathological changes in various organs and tissues. We performed transcriptome analysis to investigate the changes in potential downstream genes caused by *VASN* deficiency. Our RNA sequencing results revealed that *VASN* deficiency induced obvious changes in multiple signalling pathways, including ECM‐receptor interactions, the PPAR signalling pathway, cardiac muscle contraction and dilated cardiomyopathy (Figure 4). To the best of our knowledge, it was not known how VASN causes heart disease. Our findings indicate that the *VASN*‐knockout mouse model combined with transcriptome sequencing can be used to study the important mechanisms of organ and disease development.

Hypertrophic cardiomyopathy is the most common cause of sudden death and can lead to heart failure and stroke.^8^, ^9^, ^10^ To date, it has not been reported whether *VASN* deficiency causes cardiac hypertrophy. Our experimental results indicated that MYL7 is a possible downstream protein of VASN (Figures 3, 4 and 5). *MYL7* (MLC2a or RLCa) is an RLC family member that is expressed in both ventricles and atria.^20^, ^39^ Many studies have reported the important role of *MYL7* in early cardiac development. Deletion of *MYL7* led to abnormal atrial contraction and early embryonic death,^39^ and *MYL7* mutations resulted in myocardial dysfunction and abnormal vessel remodelling.^23^, ^39^ Moreover, *MYL7* deficiency resulted in enlarged, nonfunctional atria and secondary defects in vessel patterning,^21^ and *MYL7*‐deficient hearts showed abnormal myocardial organization and embryonic atrial function.^40^ Our results showed that MYL7 downregulation caused abnormal myocardial structure, as reported in previous studies (Figure 6). Therefore, these studies suggest that the downregulation of *MYL7* expression may lead to impaired cardiac development and damaged myocardial structure. However, the mechanism by which VASN regulates *MYL7* expression must be further explored. These results will contribute to understanding the pathogenesis of hypertrophic cardiomyopathy and identifying treatment strategies (Figure 7).

In summary, this study revealed that *VASN* plays a novel and vital role in cardiac hypertrophy. Mechanistically, we discovered that *VASN* deficiency downregulated *MYL7* expression, which induced myocardial structure abnormalities and disorders, resulting in cardiac hypertrophy. Cardiac hypertrophy may be the cause of death in young *VASN*‐knockout mice. Therefore, our results reveal a pathological phenomenon in which *VASN* deficiency may lead to cardiac hypertrophy by downregulating *MYL7* expression.

## CONFLICT OF INTEREST

All authors have no conflicts of interest.

## AUTHOR CONTRIBUTION

**Junming Sun:** Conceptualization (lead); Data curation (lead); Formal analysis (lead); Funding acquisition (equal); Investigation (lead); Methodology (lead); Project administration (lead); Resources (lead); Software (lead); Supervision (lead); Validation (lead); Visualization (lead); Writing‐original draft (lead); Writing‐review & editing (lead). **Xiaoping Guo:** Data curation (supporting); Methodology (supporting); Resources (supporting); Software (supporting); Supervision (supporting). **Ping Yu:** Conceptualization (supporting); Methodology (supporting); Project administration (supporting); Resources (equal); Software (supporting); Validation (supporting); Visualization (supporting). **Jinning Liang:** Methodology (supporting); Project administration (supporting); Resources (supporting). **Zhongxiang Mo:** Methodology (supporting); Project administration (supporting); Resources (supporting). **Mingyuan Zhang:** Formal analysis (supporting); Investigation (supporting); Visualization (supporting); Writing‐original draft (supporting). **Lichao Yang:** Conceptualization (supporting); Data curation (supporting); Project administration (supporting); Supervision (supporting); Validation (supporting). **Xuejing Huang:** Investigation (supporting); Methodology (supporting); Resources (supporting); Software (supporting). **Bing Hu:** Funding acquisition (supporting); Resources (supporting). **Jiajuan Liu:** Funding acquisition (supporting); Resources (supporting). **Yiqiang Ouyang:** Conceptualization (lead); Data curation (lead); Supervision (lead); Visualization (lead); Writing‐review & editing (lead). **Min He:** Conceptualization (equal); Data curation (lead); Funding acquisition (lead); Project administration (lead); Supervision (equal); Writing‐review & editing (lead).
